# Monte Carlo methods for medical imaging research

**DOI:** 10.1007/s13534-024-00423-x

**Published:** 2024-09-05

**Authors:** Hoyeon Lee

**Affiliations:** Department of Diagnostic Radiology and Centre of Cancer Medicine, University of Hong Kong, Hong Kong, China

**Keywords:** Monte Carlo, Medical imaging, Computational modeling

## Abstract

In radiation-based medical imaging research, computational modeling methods are used to design and validate imaging systems and post-processing algorithms. Monte Carlo methods are widely used for the computational modeling as they can model the systems accurately and intuitively by sampling interactions between particles and imaging subject with known probability distributions. This article reviews the physics behind Monte Carlo methods, their applications in medical imaging, and available MC codes for medical imaging research. Additionally, potential research areas related to Monte Carlo for medical imaging are discussed.

## Introduction

Medical images play a pivotal role in clinical settings as they provide anatomical and functional information about the human body without invasive procedures [1]. Radiation-based imaging devices, i.e., Computed Tomography (CT), Positron Emission Tomography (PET), and Single Photon Emission Computed Tomography (SPECT), are commonly used in clinical environments [2]. Recently developed imaging modalities, such as spectral CT, tomosynthesis, and breast CT, provide additional information to aid clinical decisions. Medical imaging devices have expanded their applications for image-guided radiotherapy (IGRT) for patient setup, dose verification, and treatment monitoring. This includes Cone-Beam CT (CBCT), Mega-Voltage CT (MVCT), In-beam PET, and Prompt Gamma imaging [3?^6^].

Numerous studies have been conducted to improve image quality and develop novel imaging modalities. The quality and information provided by the images are the basis of clinical decisions. Computational modeling offers safe and efficient means of obtaining realistic data and understanding radiation physics. Monte Carlo (MC) methods have been widely used in computational modeling of medical imaging studies and provide valuable insights for research and development [7].

In this article, we review the background on MC methods, their applications in medical imaging research, and commonly used codes in medical imaging research. In addition, potential research areas will be briefly discussed.

## Physics background in MC

Radiation-based medical imaging modalities employ various radiation sources depending on the purpose. For instance, X-ray CT and tomosynthesis use photons generated by X-ray tubes, PET measures photons created by the annihilation of positrons, and SPECT utilizes gamma rays produced by radioactive decay. Although these modalities involve different radiation sources, the interactions of the particles with media are governed by the Linear Boltzmann Transport Equation (LBTE) as shown in Eq. (1) [8, 9]. The equation describes the behavior of particles in a small volume $\Delta V$ around $r$. In the equation, $r$ is the position of particles, $\Omega$ is the traveling direction of particles, $E$ is the energy of particles, $\phi$ is fluence of particles, $S$ is the source term of particles, $\sigma$ is the total cross-section, and ${\sigma }_{s}$ is the differential cross-section of materials for particles.

The first term of the equation represents the fluence of particles with energy $E$ and direction $\Omega$ (($E, \Omega$)) traveling $\Delta V$ without interaction. The second term accounts for the removal of particles with ($E, \Omega$) due to absorption or scattering in $\Delta V$. The third term depicts the production of particles with ($E, \Omega$) in $\Delta V$. The fourth term describes the transition of particles from ($E{\prime}, \Omega {\prime}$) to ($E, \Omega$) in $\Delta V$.

1 $$(\Omega \cdot \nabla )\phi (r,E,\Omega )+\sigma (r,E)\phi (r,E,\Omega )=S(r,E,\Omega )+{\int }_{0}^{{E}_{0}}dE{\int }_{4\pi }d\Omega [{\sigma }_{s}(r,E{\prime}\to E,\Omega^{\prime}\to \Omega )\phi (r,E^{\prime},\Omega^{\prime})]$$

By solving the equation, one can transport particles and determine their energy, direction, and the number of particles that reach the detector.

**Fig. 1:**
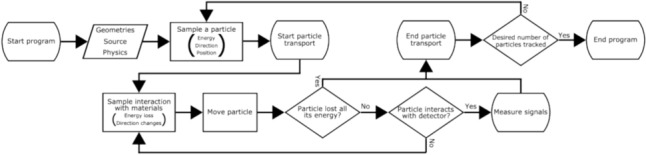
Flowchart of MC simulations

The MC methods offer stochastic solutions to the equation by tracking a multitude of particles. For a given set of geometric components, particle types, and physics data, the MC methods randomly sample interactions between particles and geometric components based on cross-section data. This determines the interaction type, scattering angle, energy loss, and secondary particle generation. The particles are transported until they lose all their energy or are captured by the detector, as shown in Fig. 1. When particles are detected, signal-processing algorithms are applied to produce image data. This process is analogous too converting incoming particles into human-readable signals in experimental systems.

**Fig. 2:**
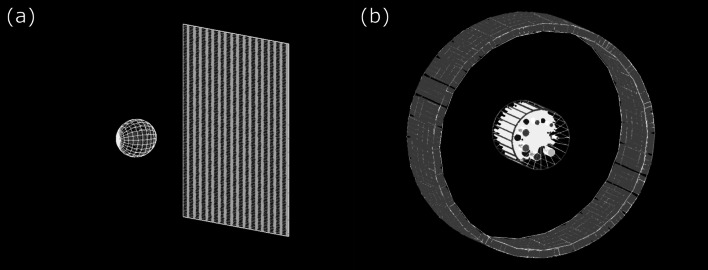
Illustration of **a** CBCT detector and Shepp-Logan phantom and **b** PET detection system and ACR-type phantom

MC methods sample particle interaction with the geometric setup of imaging objects and imaging devices. Figure 2a depicts a CBCT detector based on the Varian On-Board Imager (OBI) system [10] (Varian Medical Systems, Palo Alto, CA, USA) and a numerical 3D Shepp-Logan phantom [11]. Figure 2b shows a PET detector based on GE Discovery MI [12] and the American College of Radiology (ACR)-type phantom [13] used for quality assurance (QA) of PET systems. The geometries and phantoms are built using TOol for PArticle Simulation (TOPAS) [14, 15] and TOPAS-imaging extension [16], which will be discussed later.

The accuracy of the solutions obtained from MC methods hinges on the precise modeling of imaging systems and imaging subjects, the physics processes involved, and the number of particles tracked, which determines the statistical uncertainties of the results. Generally, achieving lower statistical uncertainty requires significant computational cost. To optimize the balance between reducing uncertainty and managing computational demands, variance reduction techniques (VRTs), i.e., Russian roulette, particle splitting, and optical spread function, are frequently utilized in MC methods [17?^20^].

## MC applications in medical imaging research

### Dosimetry

In radiation-based imaging, it is essential to estimate the potential risk to patients by calculating the radiation dose exposed during imaging [21]. Consequently, extensive studies have been conducted to develop dose calculation algorithms for imaging.

For CT dosimetry, the CT dose index (CTDI) [22] is commonly used. The CTDI calculation is based on the measurements taken with a standardized cylindrical phantom [23] made of polymethyl methacrylate (PMMA). Measurements are taken at the center and at the periphery of the phantom using an ion chamber. The weighted sum of these measurements represents the dose absorbed by patients.

For nuclear medicine, the Medical Internal Radiation Dosimetry (MIRD) [24] is used to calculate the absorbed dose from radioisotopes. The method calculates the absorbed dose to neighboring organs per radioisotope decay in a source organ, called the S-factor, using Monte Carlo for each radioisotope. The absorbed dose is then calculated by multiplying the time-integrated radioactivity of radioisotopes and S-factors.

While both CTDI and MIRD provide simplified approaches to estimate absorbed dose to patients or internal organs, patient-specific factors, i.e., patient size, and patient anatomy, should be considered for personalized dose estimation. This is particularly crucial in theragnostic applications, which involve simultaneous imaging and treatment using radiopharmaceuticals [25]. In theragnostic applications, the employed dose level (~20 Gy) is significantly higher than that used for the imaging-only purposes (~10 mSv=0.01 Gy). Therefore, accurate dose calculation for both tumors and neighboring normal organs is of utmost importance to achieve clinical goals.

To improve precision of dose calculation, the convolution/superposition methods have been developed [26?^29^]. These methods model the dose deposition of external or internal radiation sources using point-spread-kernels [30?^34^] and estimate the dose distribution through convolution and superposition operations. The point-spread-kernels, which describe the contribution of dose deposition from a source, are obtained through MC simulation to estimate the dose contribution to all potential locations from a point source.

However, the convolution/superposition algorithms typically yield higher errors with heterogeneous media [35?^38^]. Higher precision in dose calculation can be achieved by estimating the dose with MC simulation without model-based approximations. Therefore, MC-based dose calculation has been investigated for chest radiography [39, 40], CT [41?^43^], breast CT [44, 45], PET [46?^48^], SPECT [49], and digital breast tomosynthesis (DBT) [50?^52^]. MC-based dosimetry methods showed good agreement with experimental measurements, typically within 4% [53, 54].

### Image reconstruction and artifact correction

In volumetric reconstruction of medical imaging data, the system matrix is required to represent the contribution of detected signals in the image domain. The matrix maps data between image and detector domains, and its accuracy significantly impacts the image quality. MC can generate a high-quality system matrix that considers radiation physics, detector response, and particle characteristics for Tomographic Gamma Scanning [55], PET [56, 57], and SPECT [58, 59] image reconstruction.

MC methods also play a crucial role in artifact correction. Typically, image reconstruction and processing algorithms assume that the measured signals originate from mono-energetic particles and travel straight between the source and the detector. However, in reality, particles exhibit polychromatic energy distributions and can be scattered during travel due to interaction with imaging subjects. These discrepancies introduce imaging artifacts, such as scatter and beam hardening, which degrade image quality.

Scatter artifacts are common in medical imaging and significantly degrades image quality. Several scatter correction methods have been studied to remove scattered signals, including rejection methods that use a collimator or anti-scatter grid [60?^62^], beam-blocker-based estimation and removal methods [63?^65^], model-based methods [66, 67], and data-driven methods [68?^70^]. MC methods can also be used to correct for the scatter artifact by integrating MC simulation steps with image reconstruction frameworks to estimate and remove the scattered signals [71?^74^]. In such applications, fast MC methods are employed to minimize computational overhead.

Furthermore, MC methods can estimate the energy spectrum of the X-ray tube, which is necessary to account for the polychromatic nature of X-rays during image reconstruction [75]. They can also correct other imaging artifacts, including beam hardening, metal artifacts, partial volume effects, and off-focal radiation [76].

### Virtual clinical trial

The virtual clinical trial (VCT) [77?^79^], also known as an *in-silico* clinical trial, is a computational approach used to estimate the effects of treatment and medical devices by modeling human anatomy, physiology, drugs, and medical devices. Unlike traditional clinical trials, which involve subject recruitment, data acquisition, efficacy evaluation, and safety evaluation, the VCTs are time-saving and cost-effective alternatives that requires less manpower to reach conclusions.

The VCTs have also been adapted for medical imaging devices [79]. In this framework, MC plays a pivotal role as it can accurately model the imaging devices, imaging subjects, and interactions between particles and materials. Therefore, MC has been used for VCTs to validate and characterize breast imaging systems [80?^84^], develop and validate image reconstruction algorithms [85?^93^], and investigate novel imaging systems [94?^96^], and detectors [97?^99^].

Recently, the VCTs have become increasingly important in the development of data-driven algorithms due to their need for large datasets. MC methods can produce artifact-free data or filter detected signals based on the origin of generation, interaction, and particle energy. This capability allows for the generation of input data that mimics experimental measurements and paired desired output, tailored to the objectives of the study. Consequently, MC simulations are commonly used to collect data for training deep neural networks that estimate scatter artifacts [68?^70^, ^100^, ^101^], solve inverse problems for image reconstruction [102, 103], reconstruct under-sampled data [104], denoise low-dose medical images [105], and estimate dose exposure from imaging [106, 107].

## MC codes available for medical imaging research

There are several MC codes available for modeling medical imaging systems, each with its own advantages. This section covers available codes categorized into 4 groups: (1) general-purpose MC codes for experts, (2) user-friendly general-purpose codes, (3) GPU-based accelerated MC codes, and (4) commercial MC software packages.

### Geant4/Electron Gamma Shower (EGS)/FLUKA

Geant4 [108], EGSnrc [109], and FLUKA [110] are widely used MC codes for radiation transport problems. These general-purpose codes support a wide range of particle interactions, geometric components, and scoring functions. They have a broad range of applications, including medical linear accelerators (LINACs) [111?^115^], charged particle accelerators [116?^119^], and medical imaging modalities such as CT [120?^123^], PET [124?^127^], SPECT [128, 129], prompt gamma [130, 131], proton imaging [132, 133], and mammography [134].

These MC codes offer extensive customizability, allowing users to add various features, including geometric components, scoring functions, and VRTs. However, setting up the simulation environment, running simulations, and adding new features require substantial programming and physics knowledge.

### Geant4 Application for Tomographic Emission (GATE)/TOols for PArticle imulation (TOPAS))

GATE [135, 136] and TOPAS [14, 15] are general-purpose MC codes that serve as wrapper for Geant4. They support macro-based or text-based input parameters for setting up simulation environments without requiring programming knowledge. Additionally, they offer pre-built functions to facilitate simulations.

GATE is primarily designed for medical imaging device modeling, providing extensive scoring functions, geometric components, examples, and file formats commonly used in clinics, i.e., DICOM, MHA, and MHD. Numerous studies have utilized GATE to model various medical imaging systems, including CT/CBCT [93, 137?^139^], MVCT [20, 140?^142^], PET [137, 143?^147^], SPECT [137, 144, 147], prompt gamma [148, 149], and mammography [150].

TOPAS was initially designed to model proton beamlines and medical LINACs, and has recently expanded to include features for medical imaging devices through extensions [16, 151]. The TOPAS-imaging [16] extension supports detector geometries and scoring functions for CBCT, PET, SPECT, and prompt gamma imaging systems. The fastCAT [151] framework provides an integrated framework for CBCT imaging and reconstruction.

### GPU geant4-based based Monte Carlo simulation (GGEMS)/MCGPU/MCPET

The aforementioned MC codes are general-purpose and support a wide range of particles and interactions, including electrons, photons, optical photons, and charged particles. This versatility allows them to model various imaging modalities regardless of the particles, interactions, or geometries. However, they are currently only implemented on computer processing units (CPUs), which necessitates extensive computational resources for simulations, creating a bottleneck. To mitigate this computational burden, multithreading and distributed computing approaches have been widely employed [152?^155^].

Recent advancement in graphic processing units (GPUs) and programming methods have further improve computational efficiency. GPU-based MC codes such as GGEMS [156], MCGPU [83, 157, 158], and MCGPU-PET [159] transport particles in parallel to improve the efficiency. GGEMS is used for modeling CBCT and SPECT systems. MCGPU aims to model CBCT and DBT systems, and MCGPU-PET aims to model PET systems.

These GPU-based codes focus on the photoelectric effect, Compton scattering, and Rayleigh scattering of photons, interactions that are most significant for CBCT, PET, SPECT, and DBT systems. They also support geometric components and scanning geometries commonly used in medical imaging modalities. However, modifications are needed to model modalities with atypical geometries, such as non-isocentric geometry in CBCT [160].

The results obtained from the GPU-accelerated MC codes demonstrated excellent agreement with general-purpose MC codes, such as GATE [135] and PENELOPE [161, 162], with discrepancies below 1% for CBCT applications and below 10% for PET applications. Notably, the GPU-accelerated MC codes required approximately 100 times less computation time compared to the general-purpose MC codes.

### Commercial software packages

Commercial software packages supporting MC-based dose calculation for nuclear medicine, include Torch (Voximetry, Madison, WI, USA) [163] and Voxel Dosimetry (Hermes Medical Solutions, Stockholm, Sweden) [164]. These packages employ GPU-accelerated MC [165] or semi-MC [166] to calculate internal organ dose from SPECT/CT or PET/CT images within a timeframe suitable for clinical practice.

## Potential research areas

### Virtual human phantom and 4D simulation

Most studies in the VCT framework currently utilize existing patient or phantoms images to reproduce raw imaging data. The diversity of the imaging data can be further enhanced by adopting virtual phantoms that closely resemble human anatomy. Extensive studies have been conducted to develop such phantoms, including XCAT phantoms [167], NCI phantoms [168, 169], and breast phantoms [170, 171]. These phantoms account for a variety of human anatomies and include breathing and cardiac motion for realistic simulation.

Further investigation is needed to obtain more diverse and realistic images using virtual phantoms that incorporate vasculature networks, blood flow, synthetic lesions, and synthetic implants. This approach will alleviate the experimental burden in research projects and facilitate the exploration of novel methods for vascular imaging devices and 4D imaging techniques. Additionally, it can aid in the development and validation of data-driven methods by providing data that closely resembles human anatomy and disease, thereby improving the reliability of data-driven smethods.

### Benchmarking and validation datasets

Several MC codes are available for imaging research, with ongoing development to meet the specific requirements of individual research project. The physics models and results of these new MC codes have been independently validated. However, an extensive comparison between different codes has not yet been conducted. Such a comparison is important for identifying the limitations and advantages of each code and will provide essential information for researchers to choose the most appropriate MC code for their study.

A study conducted by an American Association of Physicists in Medicine (AAPM) Task Group provided detailed description of the simulation setup and datasets used to compare the half-value layer, dose exposure, and fluence of CT, radiography, and tomosynthesis systems [172]. This study also compared the results of different general-purpose MC codes. Further investigations are required to cover various imaging modalities, such as PET, SPECT, spectral CT, and MVCT, and to conduct experimental validation. Additionally, it would be desirable to establish testing criteria to compare computational costs, as was done for MC-based dose calculation comparisons [173], since most recently developed MC codes utilize advanced computational techniques and hardware to minimize computational cost.

### Deterministic solver

Although MC methods provide accurate modeling for imaging modalities, they require extensive computational resources. Consequently, numerous studies have been conducted to minimize computational costs using parallelization methods and variance reduction techniques, as previously discussed. Another approach to reducing the computational cost is to solve the LBTE deterministically.

The deterministic solution can be obtained by discretizing variables (energy, angle, and space) and employing numerical methods [174]. The deterministic approaches converge to the same solution obtained by MC codes. The uncertainty of the deterministic solution depends on the granularity of variable discretization, whereas in MC methods, it depends on the number of particles tracked.

Research into deterministic solvers has been applied to estimate scattered X-ray signals and correct them in CT images [175?^177^], as well as to estimate patient dose exposure during CT imaging [178?^180^]. While deterministic approaches have demonstrated equivalent results to MC methods [176, 180], they remain unexplored compared to MC methods. Comprehensive investigations are required to extend the deterministic solvers to other imaging modalities such as PET, SPECT, thereby enhancing their application in medical imaging research.
